# Assessing the clinical diagnostic value of anti-Müllerian hormone in polycystic ovarian syndrome and its correlation with clinical and metabolism indicators

**DOI:** 10.1186/s13048-024-01405-4

**Published:** 2024-04-10

**Authors:** Li Wang, Mengjun Luo, Xiaoyu Yu, Rong Li, Fei Ye, Dongsheng Xiong, Yan Gong, Mingyue Zheng, Weixin Liu, Jiuzhi Zeng

**Affiliations:** 1 Reproductive Medicine Center, Key Laboratory of Reproductive Medicine, Sichuan Provincial Maternity and Child Health Care Hospital, The Affiliated Women’s and Children’s Hospital of Chengdu Medical College, Chengdu, Sichuan 610045 China; 2 Department of Clinical Laboratory, School of Medicine, Chengdu Women’s and Children’s Central Hospital, University of Electronic Science and Technology of China, Chengdu, Sichuan 611731 China

**Keywords:** PCOS, AMH, Clinical diagnosis, Metabolism

## Abstract

**Background:**

This study investigated the association between Anti-Müllerian Hormone (AMH) and relevant metabolic parameters and assessed its predictive value in the clinical diagnosis of polycystic ovarian syndrome (PCOS).

**Methods:**

A total of 421 women aged 20–37 years were allocated to the PCOS (*n* = 168) and control (*n* = 253) groups, and their metabolic and hormonal parameters were compared. Spearman correlation analysis was conducted to investigate associations, binary logistic regression was used to determine PCOS risk factors, and receiver operating characteristic (ROC) curves were generated to evaluate the predictive value of AMH in diagnosing PCOS.

**Results:**

The PCOS group demonstrated significantly higher blood lipid, luteinizing hormone (LH), and AMH levels than the control group. Glucose and lipid metabolism and hormonal disorders in the PCOS group were more significant than in the control group among individuals with and without obesity. LH, TSTO, and AMH were identified as independent risk factors for PCOS. AMH along with LH, and antral follicle count demonstrated a high predictive value for diagnosing PCOS.

**Conclusion:**

AMH exhibited robust diagnostic use for identifying PCOS and could be considered a marker for screening PCOS to improve PCOS diagnostic accuracy. Attention should be paid to the effect of glucose and lipid metabolism on the hormonal and related parameters of PCOS populations.

## Introduction

Polycystic ovary syndrome (PCOS) is a prevalent endocrine disorder that primarily affect women of childbearing age and is characterized by a combination of ovulatory dysfunction, hyperandrogenism, oligomenorrhea and polycystic ovarian morphology. Its global incidence varies between around 5–10% [1]. The etiology of PCOS is multifactorial, involving genetic, endocrine, and reproductive factors and infertility [2]. Notably, approximately 50% of PCOS women are also obese and obesity exacerbates PCOS symptoms [3]. While obesity is not a prerequisite for PCOS development, clinical observations suggest that patients with PCOS, especially those who are obese often present with more pronounced symptoms, such as menstrual irregularities, infertility and miscarriages, posing challenges for clinical diagnosis and treatment [4]. Granulosa cells in preantral and small antral follicles produce anti-Müllerian hormone (AMH), a glycoprotein that belongs to the transforming growth factor-β family. In contrast to estradiol (E2) and follicle-stimulating hormone (FSH), AMH provides a superior assessment of ovarian reserve function and is unaffected by menstrual cycle fluctuations [5]. Therefore, it is important to evaluate the ovarian reservoir capacity and the primordial follicle count. As we all known, female age and accurate assessment of ovarian reserve capacity are critical to assess women’s reproductive capacity [6]. AMH and antral follicle count (AFC) are used to assess the ovarian reserve of infertility women and their response to stimulate in assisted reproductive technology (ART) [5, 7]. AMH < 1.1 ng/ml and AFC < 5–7 indicate diminished ovarian reserve (DOR) [8]. However, patients with PCOS infertility caused by anovulation, characterized by an increase in the number of AFC and an increase in ovarian reserve capacity, the decline of fertility in PCOS patients may be delayed. Therefore, it is difficult to calculate the aging of the ovary for the PCOS [9]. However, in patients with PCOS, AMH showed a high level, but it is not clear exactly the specific role of AMH in the pathogenesis of PCOS [10]. Currently, there is no international consensus on AMH diagnostic criteria for PCOS, underscoring the importance of tailored diagnostic approaches for Chinese women and the assessment of intervention outcomes during treatment.

Currently, some controversies arise regarding the clinical applications of AMH. Its levels are associated with insulin resistance and androgen levels. Various factors that influence the function of granulosa cells, such as obesity and metabolic factors, can affect AMH production [11, 12]. AMH is a sensitive serum marker in patients with PCOS, but many factors affect it, and the exact threshold has not been determined and standardized [13]. Notably, studies on hormonal and related parameters associated with PCOS, particularity those with obesity, are lacking. Early PCOS diagnosis plays a pivotal role in its clinical assessment, and patient treatment and prognosis [14]. Therefore, our study assessed the diagnostic efficacy of AMH in different subgroups of PCOS patients and the correlation between AMH and metabolic and hormone parameters was analyzed to assess. We wish to provide a basis for therapeutic interventions in PCOS.

## Materials and methods

### Study design

This study recruited 168 women diagnosed with PCOS and included 253 women as the control group by clinicians in the reproductive medicine center at Sichuan Maternal and Child Health Hospital from January 2021 to September 2023. Inclusion criteria for the PCOS group included women aged 20–37 years meeting the Rotterdam diagnostic criteria for PCOS, with at least two of the following: oligomenorrhea or amenorrhea, hyperandrogenism or clinical manifestations of hyperandrogenism, and sonographic evidence of polycystic ovarian morphology [15]. The control group comprised females aged 20–37 years with normal menstrual cycles, without ovulatory disturbances, normal basal hormone levels, and polycystic ovarian morphology in both ovaries. The exclusion criteria were (1) endocrine diseases that affect reproductive function; (2) patients with other diseases that cause hyperandrogenemia and ovulation dysfunction; and (3) use of hormone therapy in the past 3 months. Following the characteristics of the population [16], a body mass index (BMI) of ≥24 is defined as overweight or obese, and a BMI of ≥18.5 to < 24 is defined as normal for the diagnosis of obesity at childbearing age according to the BMI.

### Ethical approval

All procedures and protocols were approved by the Ethics Committee of the Sichuan Maternal and Child Health Hospital (Approval No.2021–002), and informed consent was obtained from all participating subjects.

### Laboratory tests

All venous blood samples were drawn using vacuum pipettes after 12-hours fasting period. Serum samples were collected into standard gel separation tubes for subsequent biochemical analysis. All collected samples were processed within 1 hour of collection. Fasting blood glucose (FPG), triglycerides (TG), total cholesterol (TC), low-density lipoprotein (LDL-C), and high-density lipoprotein (HDL-C) were assessed by standard laboratory techniques using Hitachi automatic analyzer (Hitachi 008AS automatic analyzer, Tokyo, Japan). Estradiol (E2), testosterone (TSTO), prolactin (PRL), progesterone (P), follicular stimulating hormones (FSH), and luteinizing hormone (LH) were quantified using standard laboratory techniques with a Mindray CL8000i automatic analyzer (Mindray, China). The concentrations of serum Anti-Müllerian Hormone (AMH) were determined ELISA kits (Kangrunbio, China). The intra-batch and inter-batch standard deviation coefficients were < 6.25% and < 8.30%, respectively.

### Statistical analysis

Data analyses were performed using SPSS 22.0 (IBM, Chicago, USA). Normally, distributed data are presented as mean ± standard deviation, while non-normally distributed data expressed as median (interquartile range [IQR]). Depending on the data’s distribution, group comparisons were conducted using either a t-test or the Mann-Whitney U test. Spearman correlation coefficients were calculated to assess the association between PCOS and AMH. Receiver operating characteristic (ROC) analysis were performed to establish the optimal cutoff values, and the sensitivity and specificity of each parameter in diagnosing PCOS were determined. Multivariate logistic regression was conducted to identify risk factors contributing PCOS development. A significance level of *P < 0.05* was considered for determining statistical significance.

## Results

### Study population

Table 1 presents the demographic information of the 421 women in the study, aged 20–37 years. The cohort comprised 168 individuals in the PCOS group and 253 in the control group. Comparative analysis showed no significant differences in age or infertility duration between the two groups. However, the PCOS group demonstrated significant higher BMI and AFC level than the control group. Additionally, the PCOS group display higher insulin levels, indicative of possible insulin resistance. Furthermore, the PCOS group exhibited significantly higher levels of LDL, TC and TG in their blood lipids compared to the control group (Table 1). A comparison of hormone indicators revealed that LH, TSTO and AMH levels were significantly higher in the PCOS group (Table 1).

**Table 1:** Baseline data of the 421 assessed women

Variables	PCOS (n = 168)	Control (*n* = 253)	*P-value*
Age (years))	29.29 ± 3.57	29.50 ± 3.67	*0.561*
Infertility age (years)	2.00 (1.00, 4.00)	2.00 (1.50, 4.00)	*0.221*
BMI(kg/m^2^)	22.85 ± 2.84	21.63 ± 2.69	*< 0.001*
FPG (mmol/L)	5.01 ± 0.49	4.91 ± 0.37	*0.655*
Insulin (μIU/ml)	10.06 ± 5.57	8.42 ± 4.89	*0.020*
HDL (mmol/L)	1.42 (1.19, 1.66)	1.51 (1.29, 1.73)	*0.537*
LDL (mmol/L)	2.51 (2.15, 2.98)	2.29 (1.94, 2.65)	*< 0.001*
TC (mmol/L)	4.59 ± 0.85	4.39 ± 0.75	*< 0001*
TG (mmol/L)	1.09 (0.79, 1.66)	0.80 (0.62, 1.12)	*< 0.001*
E2 (pg/ml)	35.44 (27.78, 44.99)	36.23 (28.40, 45.37)	*0.311*
LH (mIU/ml)	9.17 ± 5.26	4.99 ± 2.04	*< 0.001*
FSH (mIU/ml)	6.25 ± 1.59	6.41 ± 1.49	*0.293*
P (ng/ml)	0.47 ± 0.19	0.47 ± 0.19	*0.971*
PRL (μIU/ml)	318.00 (224.32, 417.33)	323.30 (257.85, 431.60)	*0.471*
TSTO (ng/ml)	0.37 (0.25, 0.51)	0.24 (0.18, 0.32)	*0.049*
AMH (ng/ml)	8.39 (6.22,12.08)	3.22 (2.07, 4.66)	*< 0.001*
AFC	28.00 (24.00, 36.00)	16.00 (12.00, 20.00)	*< 0.001*

*FPG* fasting blood glucose, *HDL* high-density lipoprotein, *LDL* low-density lipoprotein, *TC* total cholesterol, *TG* triglyceride, *E2* Estradiol, *LH* luteinizing hormone, *FSH* follicular stimulating hormone, *P* progesterone, *PRL* prolactin, *TSTO* testosterone, *AMH* anti-Müllerian Hormone, *AFC* antral follicle count

### Correlation analysis between hormone and metabolism indicators and PCOS

PCOS was positively correlated with several parameters, including FPG (r = 0.113, *P* = 0.020), insulin (r = 0.189, *P < 0.001*), BMI (r = 0.230, *P < 0.001*), AMH (r = 0.628, *P < 0.001*), LH (r = 0.474, *P < 0.001*), TSTO (r = 0.381, *P < 0.001*) and AFC (r = 0.656, *P < 0.001*). Additionally, PCOS displayed positive correlations with LDL (r = 0.195, *P < 0.001*), TC (r = 0.126, *P = 0.010*), and TG (r = 0.283, *P < 0.001*) but showed a negative correlation with HDL (r = − 0.127, *P < 0.001*). Furthermore, AMH exhibited positive correlation with LH (r = 0.460, *P < 0.001*), TSTO (r = 0.323, *P < 0.001*), AFC (r = 0.737, *P < 0.001*), LDL (r = 0.159, *P = 0.001*), TC (r = 0.125, *P = 0.010*) and TG (r = 0.167, *P = 0.001*) but demonstrated a negative correlation with FSH (r = − 0.131, *P < 0.001*).

### Independent factors predicting PCOS with hormone and metabolism indicators

After adjusting for age, the independent risk factors for PCOS were predicted using a logistic regression model, which revealed BMI, LH, TSTO, AMH, AFC and TG as independent risk factors for PCOS (Table 2).

**Table 2:** Comparison of markers of independent risk factors for PCOS

Variables	OR	95%CI	*P*
BMI	6.201	1.034–1.325	*0.013*
FBG	0.254	0.563–2.644	*0.614*
Insulin	0.545	0.963–1.087	*0.460*
LH	23.725	1.218–1.587	*< 0.001*
TSTO	14.155	10.255–1620.438	*< 0.001*
AMH	18.338	1.158–1.482	*< 0.001*
AFC	23.142	1.084–1.210	*< 0.001*
TG	15.803	1.609–4.060	*< 0.001*

*BMI* body mass index, *FPG* fasting blood glucose, *LH* luteinizing hormone, *TSTO* testosterone, *AMH* anti-Müllerian Hormone, *AFC* antral follicle count, *TG* triglyceride

### Predictive value of AMH and other indicators for PCOS

**Fig. 1:**
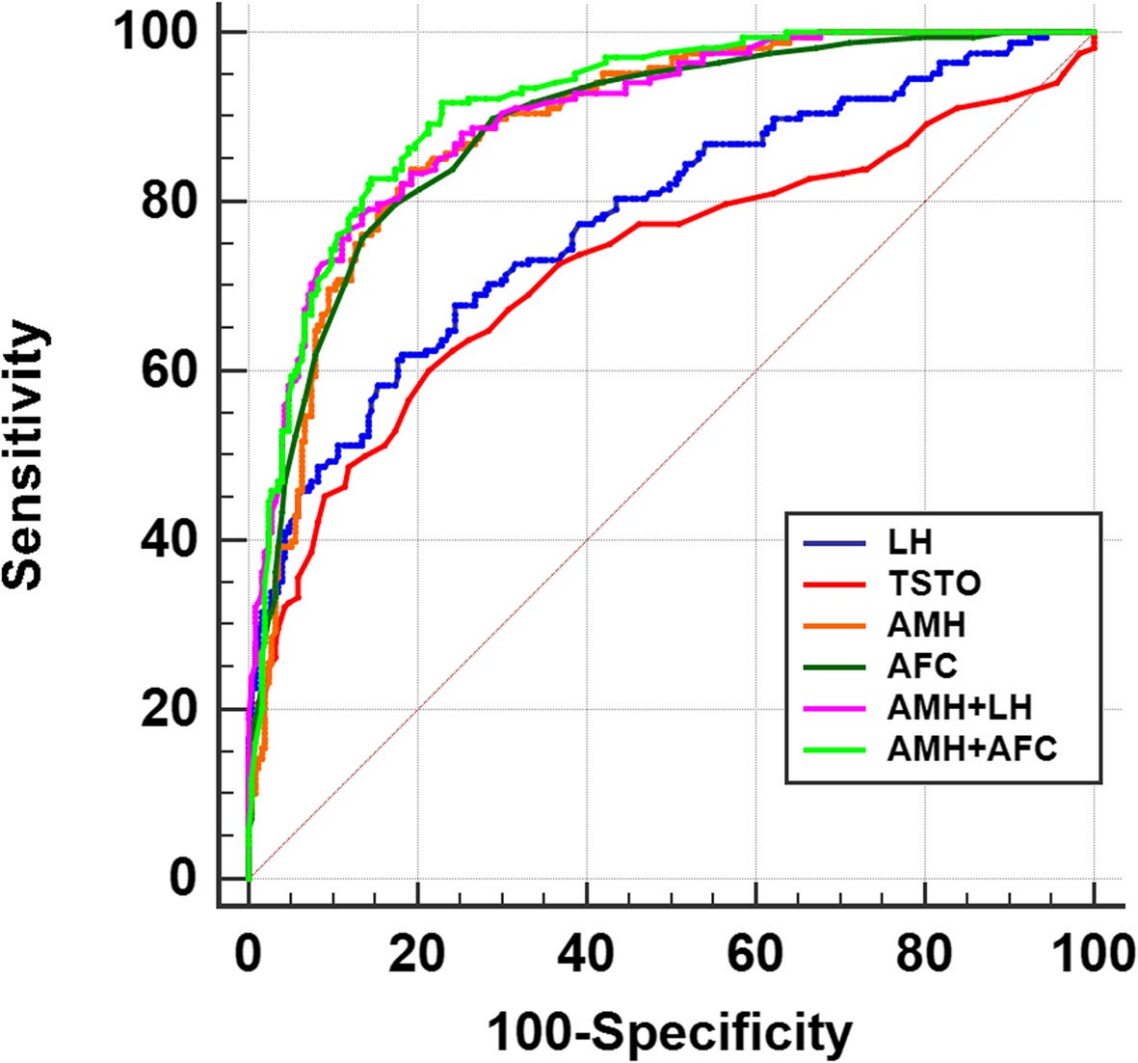
ROC curves of the predictors of PCOS of hormone indicators and AFC. LH, luteinizing hormone; TSTO, testosterone; AMH, anti-Müllerian Hormone; AFC, antral follicle count

Parameters with significant differences between the PCOS and control groups were screened, and ROC curve analysis was performed to determine their efficiency in predicting PCOS. ROC analysis results revealed that AMH exhibited the most robust predictive value, with an area under the curve (AUC) of 0.888, sensitivity of 83.93% and specificity of 80.63% (Table 3, Fig. 1). The predictive value was higher than TSTO (AUC = 0.725), LH (AUC = 0.779), and AFC (AUC = 0.887). Interestingly, the combined diagnostic value of AMH and AFC (AUC = 0.913) is higher than that of AMH and LH (AUC = 0.901) (Table 3, Fig. 1).

**Table 3:** Comparison of predictive values of AMH and other indicators of PCOS

Variables	AUC	Sensitivity (%)	Specificity (%)	Cut off value	95% CI	*P-value*
LH	0.779	61.90	81.82	6.52	0.737–0.818	*< 0.001*
TSTO	0.725	60.10	78.70	0.33	0.680–0.767	*<0.001*
AMH	0.888	83.93	80.63	5.06	0.854–0.916	*< 0.001*
AFC	0.887	79.76	82.61	22	0.852–0.915	*< 0.001*
AMH + LH	0.901	78.57	86.56	0.4	0.869–0.928	*< 0.001*
AMH + AFC	0.913	91.67	77.08	0.26	0.881–0.938	*< 0.001*

*LH* luteinizing hormone, *TSTO* testosterone, *AMH* anti-Müllerian Hormone, *AFC* antral follicle count

### Comparison of basic and hormone indexes in individual with obesity

To assess the differences in hormone and metabolic parameters among individuals with obesity (BMI ≥ 24), we conducted subgroup analyses between obese PCOS and obese control groups. We observed that levels of LH and AMH in the obese PCOS group were significantly higher (*P < 0.05*, Table 4). And the number of AFC in obese PCOS group was higher than that in obese control group (*P < 0.05*, Table 4). However, there were no significant differences in BMI, FPG, blood lipid, E2, FSH, P, PRL and TSTO between the two groups (*P > 0.05*, Table 4).

**Table 4:** Comparison of general data in the obese population

Variables	Obese PCOS (*n* = 67)	Obese control (*n* = 48)	*P-value*
Age (years)	29.74 ± 3.74	28.17 ± 3.78	*0.402*
Infertility age (years)	2.00 (1.00, 4.00)	3.00 (1.00, 3.00)	*0.732*
BMI(kg/m^2^)	25.71 ± 0.92	25.87 ± 1.67	*0.505*
FPG (mmol/L)	5.07 ± 0.51	4.92 ± 0.47	*0.065*
Insulin (μIU/ml)	11.21 ± 6.55	11.31 ± 6.09	*0.861*
HDL (mmol/L)	1.48 (1.19, 1.67)	1.37 (1.22, 1.48)	*0.124*
LDL (mmol/L)	2.51 (2.03, 3.03)	2.50 (2.22, 2.94)	*0.989*
TC (mmol/L)	4.67 ± 0.98	4.51 ± 0.69	*0.241*
TG (mmol/L)	1.15 (0.87, 1.86)	1.03 (0.75, 1.58)	*0.244*
E2 (pg/ml)	35.00 (27.27, 41.65)	32.99 (26.12, 44.28)	*0.514*
LH (mIU/ml)	8.18 ± 4.57	4.57 ± 2.21	*< 0.001*
FSH (mIU/ml)	6.09 ± 1.29	6.33 ± 1.52	*0.379*
P (ng/ml)	0.47 ± 0.19	0.42 ± 0.16	*0.188*
PRL (μIU/ml)	311.00 (209.80, 396.12)	312.20 (256.65, 398.39)	*0.408*
TSTO (ng/ml)	0.34 (0.19, 0.43)	0.26 (0.20, 0.37)	*0.115*
AMH (ng/ml)	7.98 (5.07, 10.35)	2.85 (2.01, 4.51)	*< 0.001*
AFC	26.00 (23.00, 30.00)	17.50 (12.00, 21.00)	*< 0.001*

*FPG* fasting blood glucose, *HDL*, high-density lipoprotein, *LDL* low-density lipoprotein, *TC* total cholesterol, *TG* triglyceride, *E2* Estradiol, *LH* luteinizing, *FSH* follicular stimulating hormone, *P* progesterone, *PRL* prolactin hormone, *TSTO* testosterone, *AMH* anti-Müllerian Hormone, *AFC* antral follicle count

### Predictive value of hormone-related indicators for PCOS with obesity

**Fig. 2:**
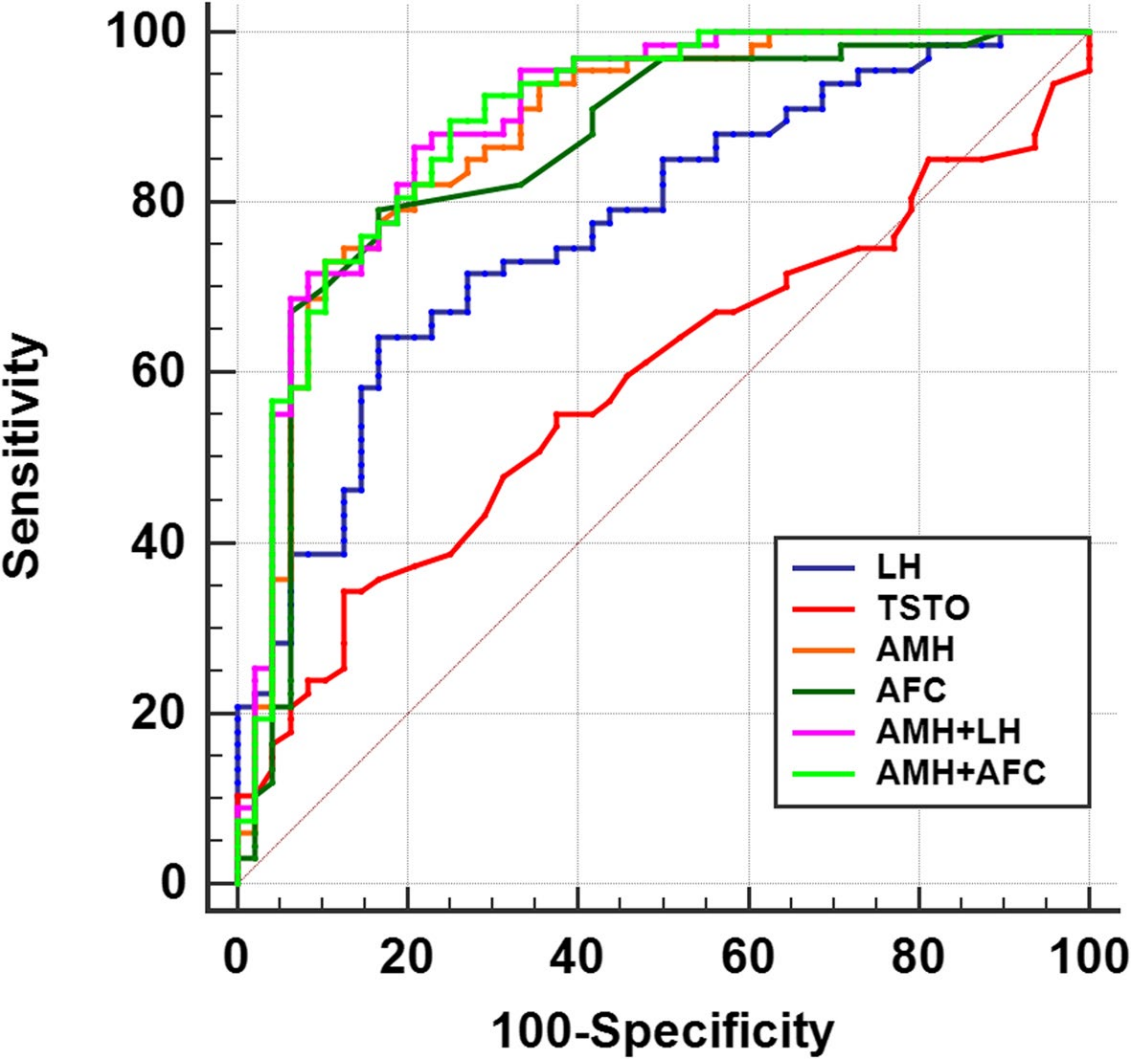
ROC curves of the predictors of hormone indicators and AFC for PCOS in the population with obesity. LH, luteinizing hormone; TSTO, testosterone; AMH, anti-Müllerian Hormone; AFC, antral follicle count

ROC curve analysis was conducted to investigate the predictive efficacy of PCOS in obese individuals. Among the individuals with obesity, AMH was found to be the most effective diagnostic parameter for PCOS (AUC = 0.879), with a sensitivity of 73.13%, specificity of 89.58%, and cut off value of 5.63 (Table 5, Fig. 2). In contrast, TSTO demonstrated a lower diagnostic capacity for PCOS (AUC = 0.587), whereas LH exhibited a certain diagnostic value for PCOS (AUC = 0.771). Remarkably, the combined diagnostic use of AMH, LH and AFC increased PCOS diagnostic accuracy. The diagnostic capacity of AMH and LH was 0.893 and that of AMH and AFC was 0.897 (Table 5, Fig. 2).

**Table 5:** Comparison of predictive value of hormones and AFC to PCOS in individuals with obesity

Variables	AUC	Sensitivity (%)	Specificity (%)	Cut off value	95% CI	*P*
LH	0.771	64.18	83.33	6.01	0.684–0.844	*<0.001*
TSTO	0.587	34.33	87.50	0.4	0.491–0.678	*0.101*
AMH	0.879	73.13	89.58	5.63	0.805–0.932	*< 0.001*
AFC	0.855	79.10	83.33	21	0.779–0.915	*< 0.001*
AMH + LH	0.893	89.55	75.00	0.42	0.811–0.936	< 0.001
AMH + AFC	0.897	86.57	79.17	0.44	0.881–0.938	< 0.001

*LH* luteinizing hormone, *TSTO* testosterone, *AMH* anti-Müllerian Hormone, *AFC* antral follicle count

### Comparison of basic and hormone indicators in individuals with no obesity

We observed no significant differences in age, years of infertility, BMI, FPG or TC levels in patients between the nonobese PCOS and nonobese control groups (*P > 0.05*, Table 6). However, nonobese PCOS group exhibited significantly elevated insulin, LDL and TG levels compared with those with the control group, and the HDL level was significantly decreased (*P < 0.05*, Table 6). The hormone levels of LH, TSTO and AMH in the PCOS subgroup were significantly higher than those in the control group. Concurrently, the number of AFCs in nonobese PCOS group was significantly higher than that in the nonobese control group (*P < 0.05*, Table 6).

**Table 6:** Comparison of general data in individuals with no obesity

Variables	Non-obese PCOS (*n* = 101)	Non-obese control (*n* = 205)	*P-value*
Age (years)	28.98 ± 3.46	29.58 ± 3.65	*0.169*
Infertility age (years)	2.00 (2.00, 4.00)	2.00 (1.75, 4.00)	*0.972*
BMI(kg/m^2^)	20.96 ± 1.95	20.64 ± 1.75	*0.147*
FPG (mmol/L)	4.95 ± 0.41	4.92 ± 0.35	*0.421*
Insulin (μIU/ml)	8.42 ± 4.89	7.74 ± 4.32	*0.011*
HDL (mmol/L)	1.40 (1.21, 1.66)	1.58 (1.34, 1.77)	*< 0.001*
LDL (mmol/L)	2.49 (2.19, 2.93)	2.23 (1.88, 2.57)	*< 0.001*
TC (mmol/L)	4.53 ± 0.75	4.35 ± 0.76	*0.057*
TG (mmol/L)	1.03 (0.75, 1.62)	0.76 (0.60, 1.03)	*< 0.001*
E2 (pg/ml)	35.77 (28.44, 46.54)	36.51 (29.20, 45.53)	*0.652*
LH (mIU/ml)	9.82 ± 5.59	5.09 ± 1.88	*< 0.001*
FSH (mIU/ml)	6.35 ± 1.76	6.43 ± 1.49	*0.682*
P (ng/ml)	0.47 ± 0.19	0.48 ± 0.19	*0.674*
PRL (μIU/ml)	337.09 (238.35, 443.85)	327.00 (258.65, 433.45)	*0.697*
TSTO (ng/ml)	0.42 (0.29, 0.54)	0.23 (0.17, 0.31)	*< 0.001*
AMH (ng/ml)	9.16 (6.92, 13.02)	3.35 (2.10, 4.77)	*< 0.001*
AFC	30.00 (24.00, 38.00)	16.00 (12.00, 20.00)	*< 0.001*

*FPG* fasting blood glucose, *HDL* high-density lipoprotein, *LDL* low-density lipoprotein, *TC* total cholesterol, *TG* triglyceride, *E2* Estradiol, *LH* luteinizing hormone, *FSH* follicle stimulating hormone, *P* progesterone, *PRL* prolactin, *TSTO* testosterone, *AMH* anti-Müllerian Hormone, *AFC* antral follicle count

### Predictive value of hormone indicators for PCOS in individuals with no obesity

**Fig. 3:**
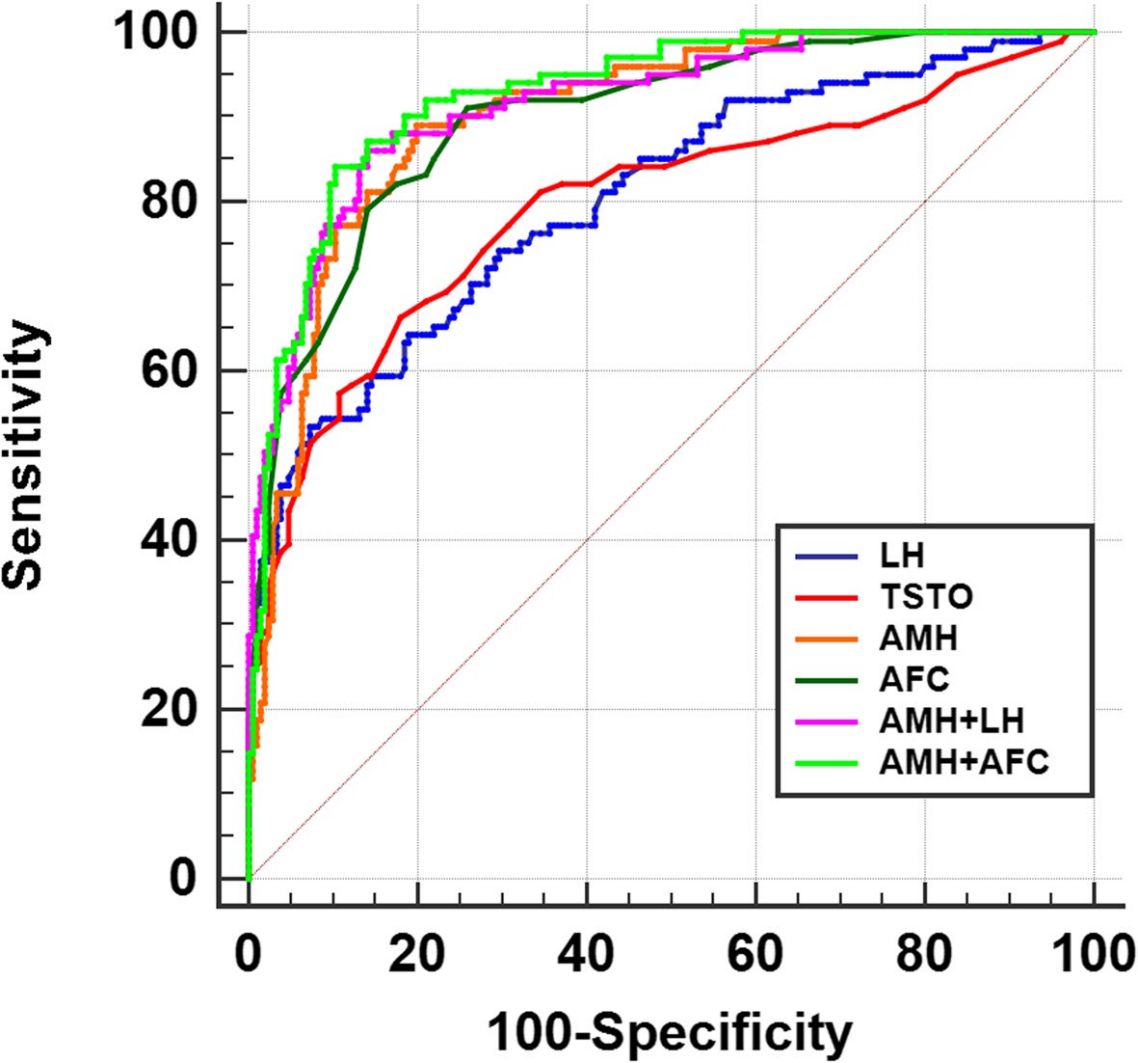
ROC curves of the predictive value of hormone indicators and AFC for PCOS with individuals with no obesity. LH, luteinizing hormone; TSTO, testosterone; AMH, anti-Müllerian Hormone; AFC, antral follicle count

In contrast to the results in obese individuals, it appears that AMH and hormone indicators had better predictive value nonobese individuals (BMI < 24). AMH exhibited the highest diagnostic value (AUC = 0.903) among LH, TSTO and AMH, (Table 7, Fig. 3). Both LH (AUC = 0.804) and TSTO (AUC = 0.796) demonstrated diagnostic utility for PCOS in individuals with no obesity. The diagnosis of PCOS frequently requires a comprehensive evaluation that includes AMH, LH and AFC in clinical practice. Accordingly, a combined diagnosis involving AMH and LH demonstrated an improved predictive value (AUC = 0.916) and AMH and AFC showed the highest predictive value (AUC = 0.927) (Table 7, Fig. 3).

**Table 7:** Comparison of the predictive value of hormones and AFC for PCOS in individuals with no obesity

Variables	AUC	Sensitivity (%)	Specificity (%)	Cut off value	95% CI	*P*
LH	0.804	53.47	92.68	7.96	0.755–0.847	*<0.001*
TSTO	0.796	66.34	81.95	0.33	0.747–0.840	*<0.001*
AMH	0.903	89.11	80.00	5.06	0.864–0.934	*< 0.001*
AFC	0.855	91.09	74.15	19	0.860–0.931	*< 0.001*
AMH + LH	0.916	86.14	85.85	0.30	0.879–0.944	< 0.001
AMH + AFC	0.927	84.16	89.76	0.36	0.892–0.953	< 0.001

*LH* luteinizing hormone, *TSTO* testosterone, *AMH* anti-Müllerian Hormone, *AFC* antral follicle count

### Comparison of hormone indicators in obese and non-obese patients with PCOS

Patients with PCOS were stratified into subgroups based on their BMI. The PCOS subgroup with obesity demonstrated notable increases in FBG levels and metabolism indicators, insulin and TG (*P < 0.05*, Table 8). In contrast, the LH, TSTO, AMH and AFC levels in the obese PCOS group were significantly decreased compared with those in the nonobese PCOS group (*P < 0.05*, Table 8).

**Table 8:** Comparison of general data for PCOS according to BMI

Variables	Obese PCOS (*n* = 67)	Non-obese PCOS (n = 101)	*P-value*
Age (years)	29.76 ± 3.71	28.98 ± 3.46	*0.166*
Infertility age (years)	2.00 (1.00, 4.00)	2.00 (2.00, 4.00)	*0.596*
BMI(Kg/m^2^)	25.71 ± 0.92	20.96 ± 1.95	*< 0.001*
FPG (mmol/L)	5.11 ± 0.57	4.95 ± 0.41	*0.036*
Insulin (μIU/ml)	10.06 ± 5.57	8.42 ± 4.89	*0.020*
HDL (mmol/L)	1.50 (1.19, 1.69)	1.40 (1.21, 1.66)	*0.457*
LDL (mmol/L)	2.51 (2.04, 3.08)	2.49 (2.19, 2.93)	*0.887*
TC (mmol/L)	4.67 ± 0.98	4.53 ± 0.75	*0.324*
TG (mmol/L)	1.15 (0.87, 1.86)	1.03 (0.75, 1.62)	*0.034*
E2 (pg/ml)	35.00 (27.27, 41.65)	35.77 (28.44, 46.54)	*0.642*
LH (mIU/ml)	8.18 ± 4.57	9.82 ± 5.59	*0.048*
FSH (mIU/ml)	6.09 ± 1.29	6.35 ± 1.76	*0.321*
P (ng/ml)	0.47 ± 0.19	0.47 ± 0.19	*0.934*
PRL (μIU/ml)	311.00 (209.80, 396.12)	337.09 (238.35, 443.85)	*0.248*
TSTO (ng/ml)	0.34 (0.19, 0.43)	0.42 (0.29, 0.54)	*0.004*
AMH (ng/ml)	7.98 (5.07, 10.35)	9.16 (6.92, 13.02)	*0.007*
AFC	26.00 (23.00, 30.00)	30.00 (24.00, 38.00)	0.024

*FPG* fasting blood glucose, *HDL* high-density lipoprotein, *LDL* low-density lipoprotein, *TC* total cholesterol, *TG* triglyceride, *E2* Estradiol, *LH* luteinizing hormone, *FSH* follicle stimulating hormone, *P* progesterone, *PRL* prolactin, *TSTO* testosterone, *AMH* anti-Müllerian Hormone, *AFC* antral follicle count

## Discussion

PCOS ranks among the most prevalent endocrine and metabolic disorders. Its etiology is believed to be multifactorial, with prior research suggesting that insulin resistance and hyperandrogenemia can be considered as its primary causative factors [17]. Additionally, some PCOS patients presented with obesity, which complicated the diagnosis process, as many clinical indicators used for PCOS diagnosis might have been influenced by various factors [18]. While AMH is less susceptible to the influence of external factors, making it an ideal marker for assessing PCOS and monitoring ovarian reserve function. Noteworthy, following the diagnostic criteria outlined in the Rotterdam criteria, certain researchers have reported elevated AMH levels exclusively in patients with type A PCOS, but not significantly increases in obese patients with type B PCOS [5, 19]. Therefore, PCOS diagnosis should simultaneously consider the effects of both body weight and metabolic patterns [20].

In patients with PCOS, the key clinical symptoms involve disruptions glucose and lipid metabolism along with hormonal abnormalities [21]. Therefore, systematically assessing the impact and correlation of relevant indicators on PCOS and AMH levels is essential. First, we analyzed fundamental metabolic and hormone-related parameters associated with PCOS. After excluding age and years of infertility as potential confounders, patients with PCOS demonstrated significantly higher body weight and insulin levels, exhibiting characteristics of obesity and insulin resistance, consistent with previous research [22]. Simultaneously, lipid metabolism indicators, including LDL, TC, and TG, were significantly increased. The significance of lipid metabolism in PCOS is frequently underestimated, and literature on this topic is limited; however, its impact on patients with PCOS is substantial [23]. PCOS displayed a significant positive correlation with TC, TG, and LDL levels. Our results indicated TG as an independent risk factor for PCOS. Previous studies considered LDL as the standard of PCOS metabolic syndrome, and lipid metabolism disorder is an important influencing factor of PCOS [24]. Hyperandrogenemia, insulin resistance, obesity, and dyslipidemia are all potential influencing factors contributing to PCOS metabolic syndrome and are the results of interaction; However, the precise mechanisms of interaction remain unclear [25]. Therefore, assessing the glucose metabolism and hormonal and lipid profiles of patients with PCOS is essential, and, when necessary, interventions should be aimed at mitigating risk factors associated with PCOS progression [26].

In most patients with PCOS, the increase in AMH levels may be attributed to follicular excess [27]. Furthermore, higher serum AMH levels are correlated with ovulation disturbance and hyperandrogenemia, indicating the potential involvement of AMH in the disturbance of follicle formation in PCOS [28]. Our study corroborated these results by confirming significantly elevated AMH, LH, and TSTO levels in patients with PCOS, all of which were determined as independent risk factors for PCOS. AMH demonstrated a positive correlation with LH and TSTO, thereby confirming the effect of the disorder of the above indicators on PCOS and a close interrelationship among AMH, LH, TSTO, and PCOS [29]. This intricate connection might be attributed the hyperinsulinemia and insulin resistance often observed in PCOS, which can stimulate TSTO production. Simultaneously, hormonal dysregulation can inhibit follicular growth and development, leading to increased AMH secretion. In turn, elevated AMH levels induce GnRH-mediated LH pulsation and secretion, further enhancing the release of ovarian androgens from the follicular membrane, thereby establishing a positive feedback loop that promotes the polycystic ovarian morphology [30]. Conversely, the high AMH of PCOS affects follicular growth by inhibiting the expression of aromatase-dependent LH receptor, which reduces the sensitivity of follicles to FSH, causing anovulation [31, 32].

In PCOS patients, obesity is prevalent, which has associated with a PCOS, incidence rate of 25.6% among obese women [33]. Obesity can lead to abnormal secretion of adipose-related factors, resulting in notable differences in serum hormone levels and related metabolic indicators compared to non-obese PCOS patients. Thus, addressing the issue of obese PCOS is of paramount concern in clinical practice [34]. Based on the above theoretical basis, we comprehensively compared patients with PCOS with and without obesity to investigate the potential for individualized diagnosis and treatment strategies. Our findings revealed that in obese women, only two hormone indicators, LH and AMH, were significantly higher levels in the PCOS group, with no substantial changes observed in lipid and glucose metabolism indicators. Conversely, in non-obese individuals, the PCOS group exhibited not only elevated levels of metabolic indicators such as insulin, LDL and TG but also higher levels of hormone indicators, including LH, TSTO and AMH. Noteworthy, the results may not align entirely with results obtained when comparing PCOS populations [1]. We also revealed that even in PCOS, individuals with no obesity demonstrated higher levels of hormone metabolism disorders, whereas individuals with obesity exhibited more significant lipid metabolism disorders. This is consistent with previous literature reports [35]. Considering the interplay between obesity and these clinical indicators, PCOS with obesity may be more easily diagnosed clinically, thereby potential overshadowing the metabolic issues frequently encountered lean PCOS cases [21].

The current diagnostic criteria for PCOS clinical practice are inconsistent. The Rotterdam criteria define PCOS based on hyperandrogenemia and the presence of polycystic ovary [15]. Previous studies revealed that the actual prevalence of PCOS might be much higher than what is clinically diagnosed [36]. In the Rotterdam standard of PCOS in 2023, the diagnostic value is affirmed. However, the cut-off value is not defined and clearly distinguished among different subgroup [37]. Previous studies revealed a close correlation between AMH and AFC and that AMH is expected to be used as a marker of ovarian reserve [38]. Its diagnostic value has been limited because of controversies regarding optimal thresholds of clinical sensitivity and specificity [39, 40]. According to different clinical manifestations, PCOS exists in patients with and without obesity, and the AMH level in patients with and without obesity is also different. Therefore, separately evaluating the diagnostic value of AMH is necessary. Herein, we evaluated the diagnostic value of PCOS using AMH, hormones, and AFC in clinical practice. Our study revealed that AMH exhibited a higher predictive value for PCOS in both individuals with and without obesity. The diagnostic cut-off values of AMH in individuals with and without obesity are 5.63 and 5.06, respectively. Combining AMH with LH and AFC for diagnosis significantly improved the diagnostic accuracy, with increased sensitivity and specificity.

Our study reveals that AMH helps in guiding diagnosis and treatment to some extent. Hormones and glucose and lipid metabolism disorders are important factors, considering the influence factors of AMH. Our present study has clarified the correlation of AMH and metabolism and hormones in individuals with PCOS with and without obesity to a certain content, but more in-depth and large-sample studies are required to further explore the exact regulatory mechanism.

## Conclusion

Our study demonstrates the clinical diagnostic value of AMH in identifying PCOS by analyzing the correlation between PCOS-related clinical metabolic indicators, hormone levels and AMH. Among PCOS patients, hyperandrogenemia and hyperlipidemia were identified as the primary manifestations, particularly in obese patients. Meanwhile, consideration the close relationship between AMH and obesity related to the disorders of glucose and metabolism, close attention should be paid to the clinical intervention and treatment of PCOS. However, due to the limitations in sample size, we could not conduct an in-depth investigation into the specific mechanisms underlying the relationship between AMH and PCOS, which could be a focus of further research efforts.

## Data Availability

The data that support the findings of this study are available from the corresponding author upon reasonable request.

## References

[1] Glueck CJ, Goldenberg N (2019). Characteristics of obesity in polycystic ovary syndrome: etiology, treatment, and genetics. Metabolism..

[2] Walter K (2022). What is polycystic ovary syndrome?. JAMA..

[3] Teede HJ, Joham AE, Paul E (2013). Longitudinal weight gain in women identified with polycystic ovary syndrome: results of an observational study in young women. Obesity..

[4] Wang Z, Groen H, Cantineau AEP (2021). Dietary intake, eating behavior, physical activity, and quality of life in infertile women with PCOS and obesity compared with non-PCOS obese controls. Nutrients..

[5] Cedars MI (2022). Evaluation of female fertility-AMH and ovarian reserve testing. J Clin Endocrinol Metab..

[6] Penzias A, Azziz R, Bendikson K (2020). Testing and interpreting measures of ovarian reserve: a committee opinion. Fertil Steril..

[7] Harris BS, Jukic AM, Truong T (2023). Markers of ovarian reserve as predictors of future fertility. Fertil Steril..

[8] Ferraretti AP, La Marca A, Fauser BC (2011). ESHRE consensus on the definition of 'poor response' to ovarian stimulation for in vitro fertilization: the Bologna criteria. Hum Reprod..

[9] Mellembakken JR, Berga SL, Kilen M (2011). Sustained fertility from 22 to 41 years of age in women with polycystic ovarian syndrome. Hum Reprod..

[10] Du J, Ruan X, Jin F (2021). Abnormalities of early folliculogenesis and serum anti-Müllerian hormone in chinese patients with polycystic ovary syndrome. J Ovarian Res..

[11] Tian X, Ruan X, Mueck AO (2014). Serum anti-Müllerian hormone and insulin resistance in the main phenotypes of non-obese polycystic ovarian syndrome women in China. Gynecol Endocrinol..

[12] Xu Y, Qiao J (2022). Association of Insulin Resistance and Elevated Androgen Levels with polycystic ovarian syndrome (PCOS): a review of literature. J Healthc Eng..

[13] Dewailly D AC, Balen A, et al. . The physiology and clinical utility of anti-Mullerian hormone in women Hum Reprod Update 2014; 20(3):370–385. 10.1093/humupd/dmt062 24430863

[14] Capuzzo M, La Marca A (2021). Use of AMH in the differential diagnosis of Anovulatory disorders including PCOS. Front Endocrinol (Lausanne)..

[15] Revised 2003 consensus on diagnostic criteria and long-term health risks related to polycystic ovary syndrome (PCOS). Hum Reprod. 2004;19(1):41–7. 10.1093/humrep/deh098 14688154

[16] Peoples Republic of China (2013). National Health Industry Standard (WS/T428–2013) adult body mass judgment[S].

[17] Chen W, Pang Y (2021). Metabolic syndrome and PCOS: pathogenesis and the role of metabolites. Metabolit..

[18] Polak K, Czyzyk A, Simoncini T (2016). New markers of insulin resistance in polycystic ovary syndrome. J Endocrinol Investig..

[19] Carmina E, Lobo RA (2022). Comparing lean and obese PCOS in different PCOS phenotypes: evidence that the body weight is more important than the Rotterdam phenotype in influencing the metabolic status. Diagnostics..

[20] Bahadur A, Verma N, Mundhra R, et al. Correlation of homeostatic model assessment-insulin resistance, anti-Mullerian hormone, and BMI in the characterization of polycystic ovary syndrome. Cureus. 2021;13(6):e16047. 10.7759/cureus.16047 PMC8321419 34336524

[21] Liou TH, Yang JH, Hsieh CH (2009). Clinical and biochemical presentations of polycystic ovary syndrome among obese and nonobese women. Fertil Steril..

[22] Bani Mohammad M, Majdi SA (2017). Polycystic ovary syndrome (PCOS) diagnostic criteria, and AMH. Asian Pac J Cancer Prev..

[23] Guo F, Gong Z, Fernando T (2022). The lipid profiles in different characteristics of women with PCOS and the interaction between dyslipidemia and metabolic disorder states: a retrospective study in Chinese population. Front Endocrinol (Lausanne)..

[24] Gambineri A, Pelusi C, Vicennati V (2002). Obesity and the polycystic ovary syndrome. Int J Obes Relat Metab Disord..

[25] Fu H, Lin Y, Deng X (2021). Correlation between anti-Mullerian hormone levels and antral follicle counts in polycystic ovary and metabolic syndromes. Syst Biol Reprod Med..

[26] Li S, Chu Q, Ma J (2017). Discovery of novel lipid profiles in PCOS: do insulin and androgen oppositely regulate bioactive lipid production?. J Clin Endocrinol Metabol..

[27] Piltonen TT, Komsi E, Morin-Papunen LC (2023). AMH as part of the diagnostic PCOS workup in large epidemiological studies. Eur J Endocrinol..

[28] Peigné M, Simon V, Pigny P (2023). Changes in circulating forms of anti-Muüllerian hormone and androgens in women with and without PCOS: a systematic longitudinal study throughout pregnancy. Hum Reprod..

[29] Barbotin AL, Mimouni NEH, Kuchcinski G (2023). Hypothalamic neuroglial plasticity is regulated by anti-Müllerian hormone and disrupted in polycystic ovary syndrome. eBioMed..

[30] Rudnicka E, Kunicki M, Calik-Ksepka A (2021). Anti-Müllerian hormone in pathogenesis, diagnostic and treatment of PCOS. Int J Mol Sci..

[31] Nardo LG, Yates AP, Roberts SA (2009). The relationships between AMH, androgens, insulin resistance and basal ovarian follicular status in non-obese subfertile women with and without polycystic ovary syndrome. Hum Reprod..

[32] Malhotra N, Mahey R, Cheluvaraju R (2023). Serum anti-Mullerian hormone (AMH) levels among different PCOS phenotypes and its correlation with clinical, endocrine, and metabolic markers of PCOS. Reprod Sci..

[33] Kataoka J, Larsson I, Björkman S (2019). Prevalence of polycystic ovary syndrome in women with severe obesity – effects of a structured weight loss programme. Clin Endocrinol..

[34] Diamanti-Kandarakis E, Dunaif A (2012). Insulin resistance and the polycystic ovary syndrome revisited: an update on mechanisms and implications. Endocr Rev..

[35] Shi W, Zhao Q, Zhao X (2021). Analysis of endocrine and metabolic indexes in non-obese patients with polycystic ovary syndrome and its compare with obese patients. Diabetes Metabol Syndrome Obes: Target Therapy..

[36] Shahid R, Iahtisham-Ul-Haq M (2022). Diet and lifestyle modifications for effective management of polycystic ovarian syndrome (PCOS). J Food Biochem..

[37] Teede HJ, Tay CT, Laven J (2023). Recommendations from the 2023 international evidence-based guideline for the assessment and Management of Polycystic Ovary Syndrome. J Clin Endocrinol Metabol..

[38] Broer SL, Broekmans FJ, Laven JS (2014). Anti-Müllerian hormone: ovarian reserve testing and its potential clinical implications. Hum Reprod Update..

[39] Iliodromiti S, Anderson RA, Nelson SM (2015). Technical and performance characteristics of anti-Müllerian hormone and antral follicle count as biomarkers of ovarian response. Hum Reprod Update..

[40] Carmina E, Campagna AM, Fruzzetti F (2016). Amh measurement versus ovarian ultrasound in the diagnosis of polycystic ovary syndrome in different phenotypes. Endocr Pract..

